# Diagnostic Performance of Dynamic Whole-Body Patlak [^18^F]FDG-PET/CT in Patients with Indeterminate Lung Lesions and Lymph Nodes

**DOI:** 10.3390/jcm12123942

**Published:** 2023-06-09

**Authors:** Matthias Weissinger, Max Atmanspacher, Werner Spengler, Ferdinand Seith, Sebastian Von Beschwitz, Helmut Dittmann, Lars Zender, Anne M. Smith, Michael E. Casey, Konstantin Nikolaou, Salvador Castaneda-Vega, Christian la Fougère

**Affiliations:** 1 Department of Nuclear Medicine and Clinical Molecular Imaging, University Hospital Tuebingen, 72076 Tuebingen, Germanymax.atmanspacher@student.uni-tuebingen.de (M.A.);; 2 Department of Diagnostic and Interventional Radiology, University Hospital Tuebingen, 72076 Tuebingen, Germany; 3 Department for Internal Medicine VIII, University Hospital Tuebingen, 72076 Tuebingen, Germany; 4 Siemens Medical Solutions USA, Inc., Molecular Imaging, Knoxville, TN 37932, USA; 5 iFIT-Cluster of Excellence, Eberhard Karls University Tuebingen, 72076 Tuebingen, Germany; 6 German Cancer Consortium (DKTK), Partner Site Tuebingen, 72076 Tuebingen, Germany; 7 Werner Siemens Imaging Center, Department of Preclinical Imaging and Radiopharmacy, Eberhard Karls University Tuebingen, 72076 Tuebingen, Germany

**Keywords:** whole-body, dynamic PET, parametric FDG, Patlak, FDG, PET/CT

## Abstract

Background: Static [^18^F]FDG-PET/CT is the imaging method of choice for the evaluation of indeterminate lung lesions and NSCLC staging; however, histological confirmation of PET-positive lesions is needed in most cases due to its limited specificity. Therefore, we aimed to evaluate the diagnostic performance of additional dynamic whole-body PET. Methods: A total of 34 consecutive patients with indeterminate pulmonary lesions were enrolled in this prospective trial. All patients underwent static (60 min p.i.) and dynamic (0–60 min p.i.) whole-body [^18^F]FDG-PET/CT (300 MBq) using the multi-bed-multi-timepoint technique (Siemens mCT FlowMotion). Histology and follow-up served as ground truth. Kinetic modeling factors were calculated using a two-compartment linear Patlak model (FDG influx rate constant = Ki, metabolic rate = MR-FDG, distribution volume = DV-FDG) and compared to SUV using ROC analysis. Results: MR-FDG_mean_ provided the best discriminatory power between benign and malignant lung lesions with an AUC of 0.887. The AUC of DV-FDG_mean_ (0.818) and SUV_mean_ (0.827) was non-significantly lower. For LNM, the AUCs for MR-FDG_mean_ (0.987) and SUV_mean_ (0.993) were comparable. Moreover, the DV-FDG_mean_ in liver metastases was three times higher than in bone or lung metastases. Conclusions: Metabolic rate quantification was shown to be a reliable method to detect malignant lung tumors, LNM, and distant metastases at least as accurately as the established SUV or dual-time-point PET scans.

## 1. Introduction

Lung cancer continues to be the tumor disease with the leading number of cancer deaths worldwide [1]. Precise staging is essential for the initiation of adequate therapy [2]. PET/CT with the glucose analog [^18^F]Fluorodeoxyglucose ([^18^F]FDG) assumes a central function for staging lung cancer, according to international guidelines [3,4]. [^18^F]FDG-PET is generally performed as a static scan, at a defined uptake time of 60 to 90 min after intravenous (i.v.) tracer application. However, due to increased [^18^F]FDG affinity in inflammatory tissue, [^18^F]FDG-PET is known to have limited specificity for an accurate evaluation of thoracic lymph nodes, especially in the presence of frequently associated tumor inflammatory pulmonary disease. Thus, [^18^F]FDG-avid lymph nodes must be biopsied before surgery or radiotherapy to rule out malignancy histologically [3,4]. However, such an intervention is often difficult and risky in clinical practice due to the often-limited cardiopulmonary reserve. Furthermore, the evaluation of indeterminate lung lesions, which cannot be biopsied due to their location or unfavorable risk–benefit to the patient, is also an indication for PET [3,4].

One way to generate complementary PET information is to quantify the tracer distribution over time. Until recently, this was typically feasible using two workflows with significant limitations. The first option is a dynamic acquisition, where the tracer distribution is continuously measured in a defined but limited anatomical region. Using this method, the axial field of view of current well-established PET scanners (generally between 15 and 30 cm) limits the anatomical coverage, which in turn restricts the dynamic acquisition of whole-body data [5,6]. A second option is a dual-/multi-time-point PET: this technique combines two or more static PET examinations and calculates the difference in [^18^F]FDG uptake [7,8,9]. Whereas traditional dynamic PET is not suitable for whole-body staging due to the limited FOV of the PET scanner, dual-time-point imaging has already shown significantly increased accuracy for the assessment of mediastinal lymph node metastases (LNM) in a large meta-analysis of 654 patients with non-small cell lung cancer (NSCLC) [7].

Dynamic whole-body PET data can be produced using an innovative combination of dynamic acquisition at the start of the scan followed by multiple subsequent whole-body scans either in the “step-and-shoot” or in the “continuous-bed-motion” technique. This form of dynamic data acquisition can be used for Patlak kinetic modeling, which enables the assessment of [^18^F]FDG distribution in different compartments separately for each organ and tissue in the body [10,11,12,13]. However, the clinical benefit of this technique and of dynamic information on tumor staging has not been completely elucidated. 

Therefore, the aim of this prospective study was to assess the feasibility of dynamic whole-body PET acquisition in a clinical setting and to evaluate the diagnostic performance of parametric imaging in the classification of indeterminate lung lesions and lymph nodes.

## 2. Materials and Methods

### 2.1. Study Design

**Figure 1:**
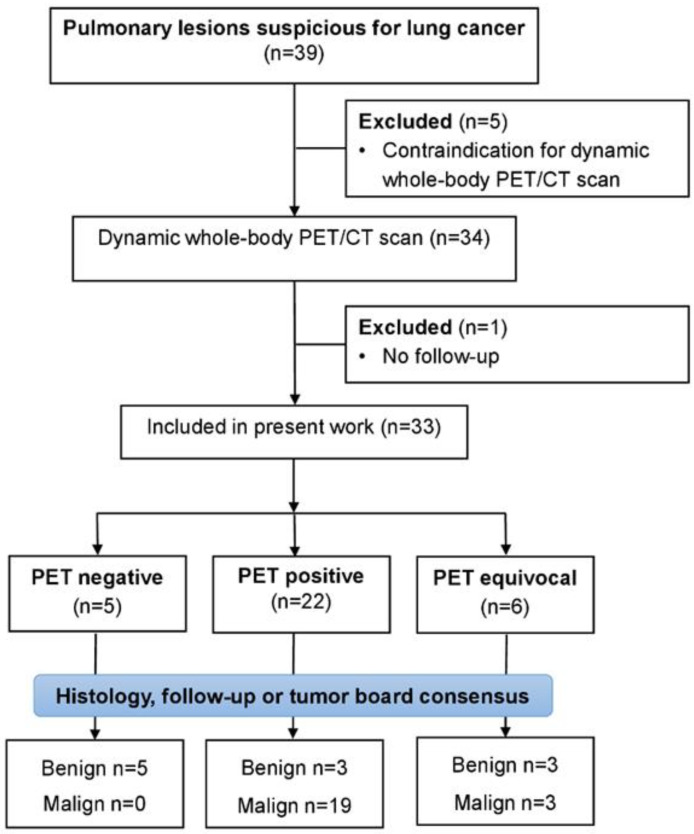
CONSORT flow diagram for patient enrolment. PET = Positron Emission Tomography; CT = Computer Tomography.

Thirty-three consecutive patients with indeterminate pulmonary lesions and a clinical indication for [^18^F]FDG-PET/CT were enrolled into this prospective unicentric trial between June 2019 and April 2022, as shown in detail in the Consolidated Standards of Reporting Trials (CONSORT) flow diagram (Figure 1). This prospective trial was approved by the Institutional Review Board (registry No. 333/2019BO2) and is listed in the German Clinical Trial Register (DRKS-ID: DRKS00017717). All patients signed an informed consent.

### 2.2. PET/CT Examination Protocol

Patients were asked to fast for at least 6 h prior to examination. Weight, size, and blood sugar level were measured before i.v. tracer administration. Blood glucose level was below 140 mg/dL in all patients without the administration of insulin 8 h prior to tracer application. [^18^F]FDG dosing was weight-based using 4.0 ± 0.6 MBq/kg. All patients were positioned with arms up on a vacuum mattress on the PET/CT (Biograph mCT, Siemens Healthineers) table to reduce motion artifacts and were asked to breathe as calmly and steadily as possible.

Before PET, a full diagnostic CT with adaptable tube voltage and tube current (CARE KV 120–140 kV, CARE Dose 4D 40–280 mAs) was performed. An iodinated contrast agent (80–100 mL Ultravist^®^ 370, Bayer Vital GmbH, Leverkusen, Germany) was administered to all patients except for contraindications.

The dynamic PET acquisition started simultaneously with the i.v. injection of [^18^F]FDG and lasted a total of 80 min. The initial table position was centered over the cardiac region (BI ≈ 6 min) to acquire the individual input function followed by whole-body (WB) dynamic PET of skull to mid-thigh (WB ≈ 74 min) using continuous-bed-motion as described in detail by Karakatsanis et al. and Rahmim et al. [10,11,12].

Image data were subdivided into 43 time frames (12 × 5 s, 6 × 10 s, 8 × 30 s, 7 × 180 s, and 10 × 300 s.) The time activity curve (TAC) was derived by an automatically generated cylindric volume of interest (VOI: 10 mm diameter and 20 mm long) centered in the descending aorta with acquired CT images using ALPHA (automated learning and parsing of human anatomy) as implemented in the vendor’s software (VG70A, Siemens Healthcare GmbH, Erlangen, Germany). 

### 2.3. Reconstruction and Postprocessing

Dynamic PET data (cardiac region and WB) were reconstructed with OSEM 3D reconstruction applying point-spread-function (PSF) and time-of-flight (TOF)—using two iterations, 21 subsets, a 200 × 200 matrix, and a 5 mm Gaussian filter. The reconstructed passes 12–17 of the WB and the resulting TAC were used to perform the Patlak reconstructions with two iterations, 21 subsets, a 200 × 200 matrix, and a Gaussian 5 mm filter as implemented in the vendor’s software (VG70A, Siemens Healthcare GmbH). 

A standard of care static whole-body image was reconstructed by using passes 15–17 of the WB, with ultraHD-PET (PSF + TOF), two iterations, 21 subsets, and a 400 × 400 matrix with a Gaussian 2 mm filter. 

[^18^F]FDG kinetics were modeled using a two-compartment model based on linear Patlak analysis [14,15], as described in detail by A. M. Smith et al. [16], resulting in the generation of whole-body Patlak slope and Patlak intercept parametric images. Patlak slope, which represents the constant influx rate of [^18^F]FDG (Ki_mean_, given in mL/(min × 100 mL) = 0.01 × min^−1^), was multiplied by the blood glucose level to calculate the metabolic rate of [^18^F]FDG (MR-FDG_mean_) and is expressed as µmol/(min × 100 mL). Patlak intercept is expressed in percent and represents the distribution volume of free [^18^F]FDG (DV-FDG_mean_) in the reversible compartments and fractional blood volume [13]. Semiquantitative measurements were performed in static images using SUV_max_, SUV_mean_ (50% isocontour), and SUV_peak_ (1 mL sphere).

### 2.4. Image Evaluation and Segmentation

Parametric images were produced and quantified using syngo.via^®^ 8.2 (Siemens Healthineers, Erlangen, Germany). Volumes of interest (VOIs) were manually delineated in the fused PET/CT images and validated by a certified expert in nuclear medicine with more than five years of experience in PET/CT. VOIs were overlaid on the Ki dataset, DV-FDG, and on the static PET images for data extraction. If necessary, manual coregistration was performed to assure adequate realignment.

### 2.5. Ground Truth

The final diagnosis was provided by histology, long-time follow-up, and/or as a consensus decision of the institutional interdisciplinary tumor board. 

### 2.6. Statistical Analysis

Differences in the mean values of two groups, features, or methods were tested for significance using the two-sided Student’s *t*-test. Levene’s test was performed to assess the equality of variance before the *t*-tests.

One-way ANOVA was performed to compare the dignity (inflammation, benign, or malign) of the different groups for the studied metrics (e.g., DV-FDGmean, MR-FDGmean). An alpha level of 0.05 was used for analysis. Subsequent multiple comparison correction was performed using Tukey’s honestly significant difference procedure. Results of the ANOVA are shown with *p* values in the main manuscript. Correlation coefficients were calculated according to Pearson and a Pearson correlation coefficient of r > 0.7 was defined as strong, 0.7–0.3 as moderate, and <0.3 as a weak linear correlation. A *p*-value < 0.05 was considered statistically significant.

The intersection of the false-negative and false-positive rates was defined as the optimal cut-off value. Statistical analysis was performed with SPSS Statistics 28.0 software (IBM Inc., Armonk, NY, USA), MATLAB v. R2022b (The MathWorks, Inc., Natick, MA, USA), and MS Excel 2019 v.2206 (Microsoft corporation, Redmond, WA, USA).

## 3. Results

### 3.1. Patient Cohort

Thirty-nine patients met the inclusion criteria for this prospective study between October 2019 and April 2022, of whom 34 consented to study-related dynamic PET acquisition. One patient received further treatment abroad and dropped out of the analysis. Consequently, 33 patients with complete datasets were included in the analysis. Gender distribution was 42% women (14/33) and 58% men (19/33). Male patients were significantly older (68 ± 9 yrs vs. 60 ± 10 yrs, respectively, *p* = 0.032) and taller (178 ± 9 cm vs. 161 ± 9 cm, respectively, *p* < 0.001) than female patients with comparable weight (78 ± 22 kg vs. 70 ± 10 kg, respectively, *p* = 0.053) and BMI (26 ± 6 vs. 27 ± 4, respectively, *p* = 0.475). The blood glucose level before tracer administration did not differ between the sexes and was 5.44 ± 0.94 mmol/L. 

### 3.2. Pulmonary Lesions

Detailed pulmonary lesion analysis is shown in Table 1 with 66.7% (22/33) classified as malignant and 33.3% as benign. In one patient, the lung lesion had completely regressed between external CT-scan and PET/CT, so that no lung lesion measurements could be obtained. The final diagnosis was confirmed histologically in 64.6% of the patients (21/33), by follow-up in 21.2% (7/33), and as a consensus decision of the interdisciplinary tumor board in 15.2% (5/33).

### 3.3. Feasibility of Patlak-PET Data Acquisition

**Figure 2:**
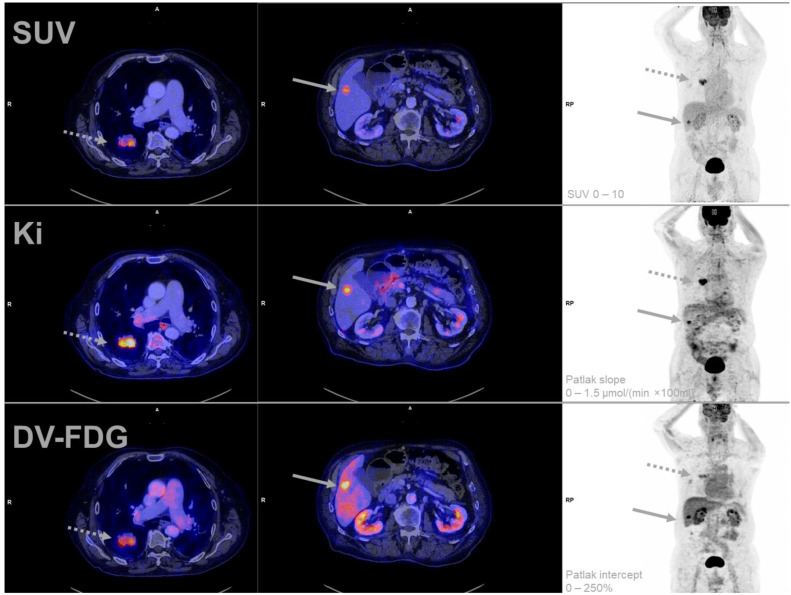
Representative example of multiparametric [^18^F]FDG PET-imaging of a patient (Study-ID 33) suffering from an adenocarcinoma of the lung (dotted arrow). A single liver metastasis was detected with PET and was histologically confirmed (solid arrow). Of note is the high DV-FDG of the liver metastasis compared to the lung tumor in combination with homogeneous imaging of the surrounding tumor-free liver parenchyma. DV-FDG = Distribution Volume of FDG; FDG = Fluorodeoxyglucose; Ki = Influx Rate Constant; PET = Positron Emission Tomography; SUV = Standardized Uptake Value.

All patients tolerated the complete scheduled acquisition time. No examination had to be discontinued or repeated due to technical difficulties. A representative multiparametric scan is presented in Figure 2.

### 3.4. Effect of Quantification Method on Diagnostic Accuracy

Each semiquantitative PET measurement was performed using three different quantification methods: max, mean (50% isocontour), and peak (1 mL sphere). The quantification method showed no significant effect on the AUC, neither for the lung lesions nor for the lymph nodes, as detailed in Supplementary Tables S1 and S2. For clarity, only the “mean” value is reported in the results. 

**Figure 3:**
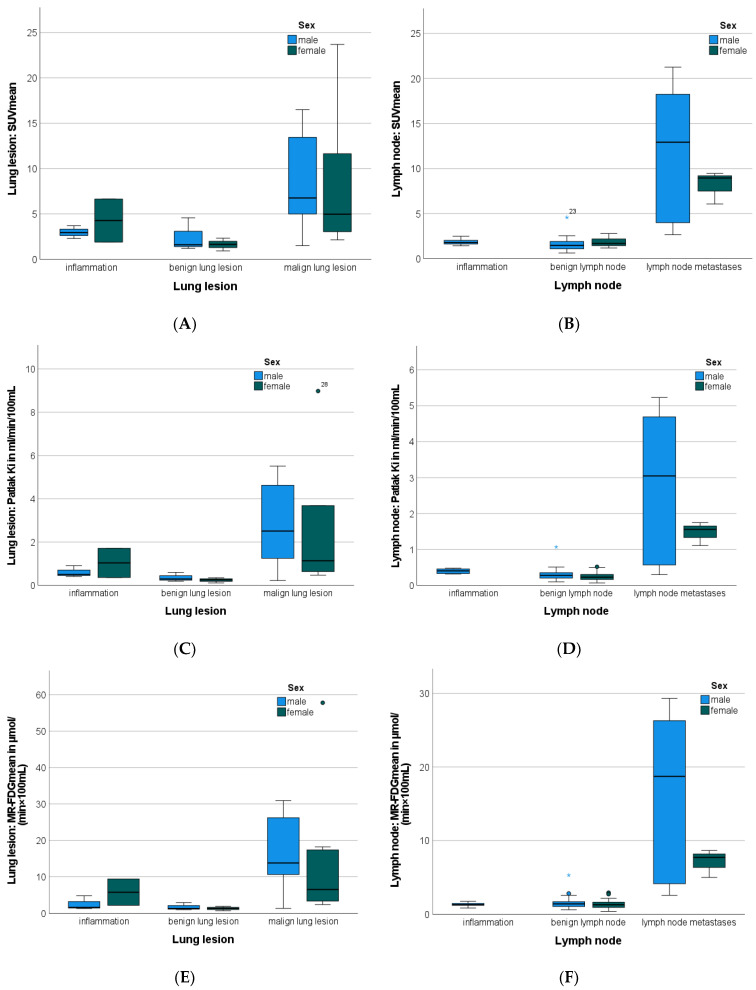

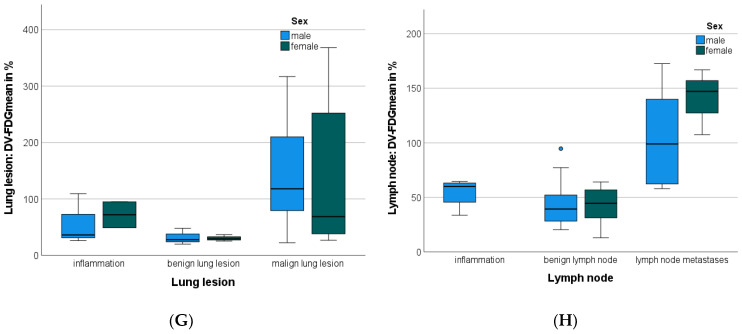
Boxplots illustrating gender-specific SUV_mean_ (**A**,**B**) Patlak Ki_mean_ (**C**,**D**) MR-FDG_mean_ (**E**,**F**) and DV-FDG_mean_ (**G**,**H**) measurements in the function of lung lesions (**A**,**C**,**E**,**G**) and lymph nodes (**B**,**D**,**F**,**H**). Asterisk (⋆) represents an extreme value. Circle (o) represents an outlier. DV-FDG = Distribution Volume of FDG; FDG = Fluorodeoxyglucose; Ki = Influx Rate Constant; MR = Metabolic Rate; PET = Positron Emission Tomography; SUV = Standardized Uptake Value.

Malignant lung lesions revealed a significantly higher tumor volume, SUV_mean_, Patlak Ki_mean_, MR-FDG_mean,_ and DV-FDG_mean_ compared with benign lung lesions, as detailed in Table 2 and Figure 3. Benign pulmonary nodules were markedly smaller than inflammatory sites, however, this difference was not significant in this cohort (*p* = 0.057). 

### 3.5. Lymph Nodes Characteristics

LNM had a significantly higher SUV_mean_, Patlak Ki_mean,_ MR-FDG_mean,_ and DV-FDG_mean_ compared to benign and to inflammatory altered LN. Furthermore, LNM presented a significantly larger short- and long-axis diameter compared to benign and to inflammatory-altered LN, as presented in Table 2**.** Tumor volume was not a feature that was consistently increased in malignant lesions and could, therefore, not significantly discriminate dignity between the three groups in this cohort. 

### 3.6. Patlak FDG-PET: Dynamic Parameter Evaluation

Liver tissue was chosen as the reference organ and measurements were performed in all patients (n = 33) in tumor-free liver tissue (SUV_mean_: 2.79; MR-FDG_mean_: 2.08 µmol/(min × 100 mL); Ki_mean_: 0.406 mL/(min × 100 mL).

**Figure 4:**
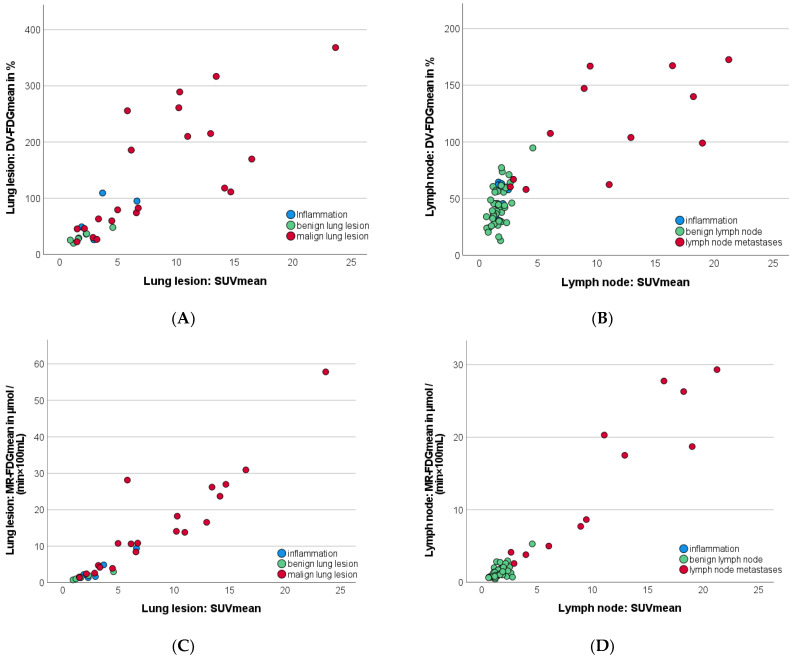
Scatterplots illustrating the correlation between SUV_mean_, MR-FDG_mean,_ and DV-FDG_mean_ of different types of lung lesions (**A**,**C**) and lymph nodes (**B**,**D**). Interestingly, DV-FDG_mean_ (**B**) and MR-FDG_mean_ (**D**) of the lymph nodes were proportionally half of the values of primary lesions (**A**,**C**), while the magnitude of SUV_mean_ of lymph nodes and primary lesions was found similar. DV-FDG = Distribution Volume of FDG; FDG = Fluorodeoxyglucose; Ki = Influx Rate Constant; MR = Metabolic Rate; PET = Positron Emission Tomography; SUV = Standardized Uptake Value.

Ki_mean_ and MR-FDG_mean_ correlated strongly for lung lesions (r = 0.989; *p* < 0.001) and LN (r = 0.994; *p* < 0.001), so that only MR-FDG_mean_ is shown in the following figures for reasons of conciseness. Quantified MR-FDG_mean_ correlated strongly with SUV_mean_ for lung lesions (r = 0.930; *p* < 0.001) as well as LN (r = 0.967; *p* < 0.001), as presented in Figure 4. The correlation between DV-FDG_mean_ and MR-FDG_mean_ was slightly lower but still strong and significant (lung lesions: 0.826, LN: 0.760, *p* < 0.001).

**Figure 5:**
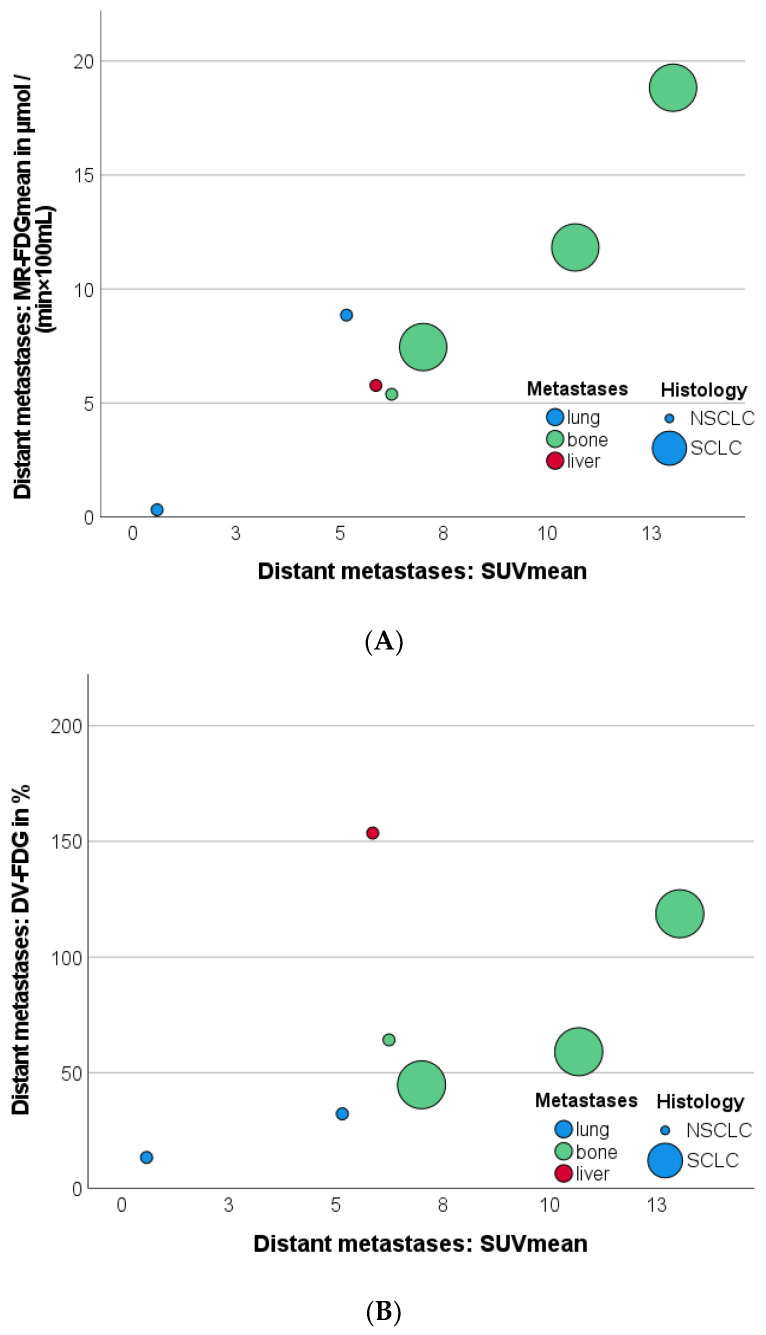
Scatterplots illustrating the correlation between SUV_mean_ and MR-FDG_mean_ (**A**); and SUV_mean_ and DV-FDG_mean_ (**B**) measurements in the function of the type of distant metastases and primary tumor histology. Metastases of NSCLC are coded as small circle, SCLC as large circle. DV-FDG = Distribution Volume of FDG; FDG = Fluorodeoxyglucose; MR = Metabolic Rate; NSCLC = Non Small Cell Lung Cancer; SUV = Standardized Uptake Value; SCLC = Small Cell Lung Cancer.

In distant metastases, MR-FDG_mean_ quantification showed a strong correlation (r = 0.943; *p* < 0.001) with SUV_mean_, regardless of the location of metastases or histology of primary tumors, as presented in the scatterplot in Figure 5A.

When only bone and lung metastases were considered, a strong correlation between SUV_mean_ and Patlak intercept was observed (r = 0.891; *p* = 0.017).

In contrast, DV-FDG_mean_ revealed a three-times higher value in an NSCLC liver metastasis (153.63%) compared to the other bone and lung metastases (55.54%), as shown in Figure 5B. As a result, the correlation with SUV_mean_ fell below the significance level (r: 0.457, *p* = 0.302). However, considering only bone and pulmonary metastases, a strong correlation between SUV_mean_ and DV-FDG_mean_ r = 0.891 (*p* = 0.017) was found.

### 3.7. Discriminatory Power between Benign and Malignant Lung Lesions

**Figure 6:**
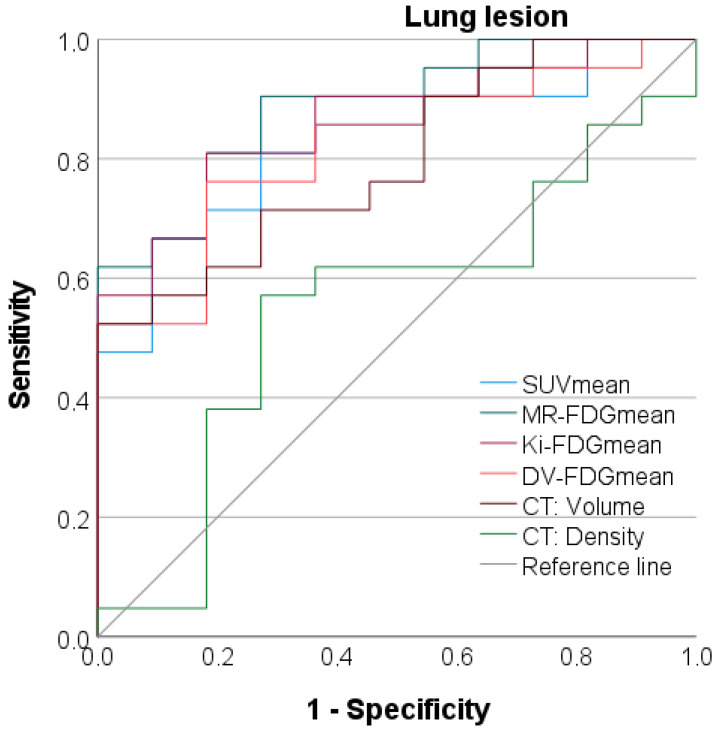
ROC analyses of CT morphologic, static as well as parametric, PET data to differentiate between malignant and benign lung lesions. CT = Computer Tomography; DV-FDG = Distribution Volume of FDG; FDG = Fluorodeoxyglucose; Ki = Influx Rate Constant; MR = Metabolic Rate; NSCLC = Non Small Cell Lung Cancer; SUV = Standardized Uptake Value; SCLC = Small Cell Lung Cancer.

SUV_mean_ and the dynamic parameters Patlak Ki_mean_, MR-FDG_mean,_ and DV-FDG_mean_ revealed very good discriminatory power in the AUC-analysis between benign and malignant lung lesions even at high-significance levels (*p* < 0.001), as detailed in Figure 6 and Table 3.

MR-FDG_mean_ provided the best discriminatory power between benign and malignant lung lesions with a high AUC of 0.887. At a somewhat lower level, the AUC of DV-FDG_mean_ was 0.818 and that of the SUV_mean_ was 0.827, although the difference did not reach significance in the AUC comparison in this cohort. MR-FDG_mean_ was slightly more specific than SUV_mean_ (81.8% vs. 72.7%, respectively) at a sensitivity of 81.0% (cut-off value of 61.7 µmol/(min × 100 mL)).

Normalizing the SUV_mean_ of the lung lesions to the SUV_mean_ of the blood pool in the descending aorta or the hepatic parenchyma did not result in a relevant AUC improvement, as presented in Table 3.

Regarding CT features, malignant lung lesions presented with significantly larger volume, as detailed in Table 2. Determination of the pulmonary nodule density was not able to reliably distinguish tumor foci from benign lung lesions (*p* = 0.65).

### 3.8. Discriminatory Power between Benign and Malignant Lymph Nodes

**Figure 7:**
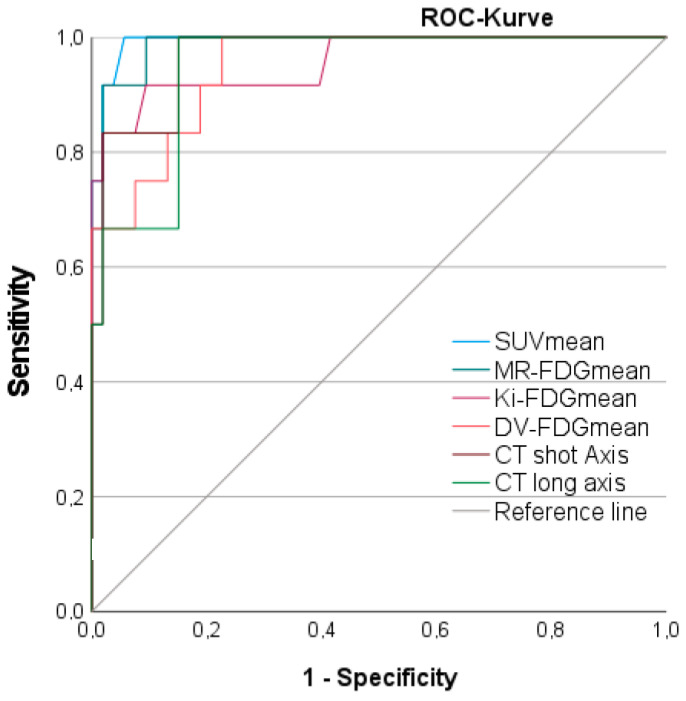
ROC analyses of CT morphologic, static as well as parametric, PET data to differentiate between malignant and benign lymph nodes. CT = Computer Tomography; DV-FDG = Distribution Volume of FDG; FDG = Fluorodeoxyglucose; Ki = Influx Rate Constant; MR = Metabolic Rate; NSCLC = Non Small Cell Lung Cancer; SUV = Standardized Uptake Value; SCLC = Small Cell Lung Cancer.

The parametric PET parameters MR-FDG_mean_, Patlak Ki_mean,_ and DV-FDG_mean_ provided excellent discriminatory power between LNM and benign LN. The AUC of the static PET parameter SUV_mean_ (AUC 0.993) was slightly, but not significantly, higher than parametric PET parameters, as detailed in the ROC (Figure 7) and Table 4. SUV_mean_ showed the highest sensitivity and specificity within all PET parameters at an optimal cut-off value of SUV 2.6.

For parametric PET, MR-FDG_mean_ revealed the highest AUC of 0.987 followed by Patlak Ki_mean_ and DV-FDG_mean_ with non-significantly lower AUC of 0.958 and 0.948, respectively. Semiautomatic diameter measurements also reached excellent AUC with 0.969 for the short-axis and 0.947 for the long-axis diameter, as shown in Figure 7 and Table 4. The calculation of the tumor-to-liver or tumor-to-metastases ratios did not improve AUC for either Patlak Ki_mean_, MR-FDG_mean_, DV-FDG_mean,_ or SUV_mean_.

### 3.9. Effect of Distant Metastases on SUV_mean_, Patlak Ki_mean_, and DV-FDG_mean_ Values of Primary Tumor and LNM

A further analysis was performed to assess the differences in SUV, Patlak Ki, MR-FDG_mean_, and DV-FDG_mean_ of lung lesions and LNM in patients with or without distant metastasis (M1, contralateral thoracic and/or extrathoracic). LNM presented with significantly higher SUV_mean_ (M1: 13.49 ± 5.65; M0: 3.89 ± 1.89 *p* = 0.018), Patlak Ki_mean_ (M1: 3.09 ± 1.63; M0: 0.63 ± 0.43 mL/min/100 mL, *p* = 0.031), and MR-FDG (M1: 17.78 ± 9.31; M0: 3.90 ± 1.22 µmol/(min × 100 mL), *p* = 0.032), but non significantly higher DV-FDG_mean_ (M1: 124.16% ± 44.78; M0: 78.23 ± 25.55, *p* = 0.129) values in patients with distant metastases (n = 5) compared to M0.

However, primary tumors showed only non-significantly higher SUV_mean_ (10.33 ± 5.37 vs. 5.73 ± 5.37%), Patlak Ki_mean_ (3.2 ± 1.85 vs. 1.69 ± 2.1 mL/min/100 mL), MR-FDG_mean_ (18.23 ± 11.01 vs. 9.45 ± 12.60 µmol/(min × 100 mL)), and DV-FDG_mean_ (143.11 ± 91.01 vs. 104.28 ± 101.48%) values in patients with M1 compared to M0.

## 4. Discussion

This prospective study investigates the additional diagnostic value of whole-body parametric Patlak analysis of [^18^F]FDG PET in patients with indeterminate lung lesions in a clinical setting. Moreover, we explore the diagnostic performance of dynamic data in the detection of LNM and distant metastases compared to standard static PET scans at 60 min p.i. First, methodologically, we demonstrate the reliability of dynamic whole-body PET/CT acquisition in a multi-bed–multi-timepoint technique with continuous table movement in the clinical routine on a conventional PET scanner. Second, we confirm that the quantified metabolic rate of [^18^F]FDG (MR-FDG) seems to be at least as accurate in distinguishing malignant from benign findings as the state-of-the-art semiquantitative SUV measurement using 60 min p.i. static scan.

Parametric data from MR-FDG and Patlak Ki correlated strongly with the established SUVmean measurements and had comparable AUCs for the classification of lung lesions. However, a closer look at the ROC indicated a slightly higher specificity in the mid-high sensitivity range for MR-FDG. This finding may indicate that MR-FDG and Ki are slightly more robust than SUV, which is in line with the results of the virtual clinical trial by Ye et al. [17]. In that study, the Ki was found to be superior to the SUV in the detection of NSCLC and more robust in the case of significant count rate reductions. However, the findings were validated only on a small sample size [17].

The parametric whole-body dynamic [^18^F]FDG PET measurements of our trial were consistent with the limited data available from previous studies [18]. In direct comparison to single-bed dynamic PET measurements published by Yang et al., our results demonstrate slightly higher SUVs in the primary tumor (M0: SUV_mean_ 5.73 vs. 5.23; M1: 10.33 vs. 8.41), and considerably lower Ki values (M0: 0.0169 min^−1^ vs. 0.026; M1: 0.032 min^−1^ vs. 0.050) [6]. Similar results were also found for LNM, whose uptake was also shown to be dependent on the presence of distant metastases (SUV_mean_: M0: 3.89 vs. 4.22; M1: 13.49 vs. 5.57) [6].

While SUV_mean_ measurements are generally accepted in the clinical setting, the use of Ki_mean_ is not validated yet. Here, the MR-FDG values of the lung tumors differed up to a factor of two compared to the dynamic single-bed measurements at comparable SUV_mean_. This effect was more emphasized and indeed dependent on the presence of distant metastases (Patlak Ki_mean_: M0: 0.0063 vs. 0.016 min^−1^, M1: 0.031 vs. 0.033 min^−1^) [6]. Notably, our data showed a significantly stronger correlation between SUV_mean_ and Patlak Ki_mean_ (r: 0.93–0.97 vs. 0.76–0.88) compared to the data published by Yang et al. [6]. Such varying strength of correlation between two parameters, which were calculated at one site each, indicate that the Ki values may depend on the calculation method. However, this must be further investigated.

In addition, it is also important to consider that although the magnitude increments of SUV_mean_ and Patlak Ki_mean_ or MR-FDG_mean_ are quite similar, they represent different physiological information. SUV_mean_ is the sum of metabolized [^18^F]FDG-6P trapped in the compartment and un-metabolized [^18^F]FDG, while MR-FDG solely reflects metabolized [^18^F]FDG-6P activity [18].

Furthermore, data on our DV-FDG measurements, which represents the combined distribution volume of free [^18^F]FDG in blood and tissue (reversible compartment), also revealed strong correlations with trapped [^18^F]FDG measured within MR-FDG and Patlak Ki_mean_ (irreversible compartment) [18]. Interestingly, the only hepatic metastasis in our cohort was visually more distinct and focal in the parametric DV-FDG image, compared to the other parametric parameters. Furthermore, this lesion presented with a remarkably higher DV-FDG value, when compared to the lung or bone metastases. One potential explanation for this effect in the liver metastasis is a previously reported increment of dephosphorylation of the trapped [^18^F]FDG-6P in liver tissue [18]. High dephosphorylation activity would result in less irreversible trapping and significant efflux of the initially trapped [^18^F]FDG-6P via the bidirectional GLUT (esp. GLUT 1) transporter out of the cell and back into plasma [18]. This would result in higher DV-FDG values since the reversible compartment also includes both free [^18^F]FDG in blood and tissue as well as some [^18^F]FDG-6P [18]. Even if the value of DV-FDG has caused some controversy [19], our data are supportive of investigations evaluating DV-FDG as a potential imaging biomarker for liver metastases.

Interestingly, in our cohort, the diagnostic performance of Patlak Ki_mean_ and MR-FDG seems to achieve at least equal or higher discriminatory power in the detection of mediastinal LNM when compared to the dual-time-point (DTP) dynamic PET using an SUV retention index (RI-SUV) between 1 h and 2 h p.i. by Shinya et al. [9] or the DTP data presented in the largest meta-analysis by Shen et al. [7] (AUC 0.958 vs. 0.794 and 0.9331) on lesion-based analysis. In detail, our MR-FDG_mean_ quantifications presented with higher sensitivity of 92% vs. 74% at a defined specificity of 76% and higher specificity of 89% vs. 76% at a defined sensitivity of 74% compared to the DTP-based RI-SUV estimation published by Shinya et al. [9].

Regarding the performance of dynamic parameters for the detection of distant metastases, there are still insufficient data in the literature. The parametric [^18^F]FDG dynamic data presented in this study, however, provide the largest published cohort with histologic validation. MR-FDG was shown to be a robust parameter with a very strong correlation to SUV_mean_ regardless of the histology of the primary tumor or location of metastasis (bone, lung, or liver).

### Limitations

There are several limitations in this prospective pilot study. First, the sample size of LNM and distant metastases is relatively small, even though it represents one of the largest published collectives. However, due to large effect sizes, the data presented are significant and, therefore, might enable a pre-conclusive analysis.

In addition, some of the lesions could not be confirmed by biopsy; thus, the diagnosis had to be confirmed based on the conclusion of the interdisciplinary tumor board, as is the gold standard for many lesions.

Data acquisition was performed within a single-center study setting; thus, the intercomparability of measurements between different PET scanners cannot be evaluated.

## 5. Conclusions

The dynamic whole-body acquisition of [^18^F]FDG using the Patlak plot was shown to be a stable method for the determination of whole-body glucose metabolism dynamics that operates well in routine clinical practice even on a standard PET/CT scanner. The quantification of the MR-FDG detects malignant lung tumors, LNM, and distant metastases with at least comparable accuracy as the established SUV_mean_ or time-consuming dual-time-point PET scans. In contrast to MR FDG, which correlates strongly with SUV, the distribution volume (DV) of [^18^F]FDG was considerably higher in liver metastases, indicating a potential additional benefit for the Patlak parameter DV-FDG in detecting hepatic metastases.

## Figures and Tables

**Table 1:** Patients’ characteristics and diagnosis.

Study-ID	Sex	Age at PET	Final Diagnosis of Lung Lesion	Diagnosis Confirmation	Tumor Stage		
					T	N	M
1	f	54	Inflammation	Follow-up			
2	m	81	CLL	Biopsy			
3	f	56	Benign	Follow-up			
4	m	75	NSCLC	Surgery	T4	N2	M1a
5	m	61	Hematoma	Follow-up			
6	m	58	NSCLC	Surgery	pT3	pN0	cM0
7	m	64	Inflammation	Follow-up			
8	m	78	NSCLC	Biopsy	cT3	cN2	cM0
9	f	50	NSCLC	Biopsy	cT4	cN3	cM1
10	m	82	Benign	Follow-up			
11	m	66	NSCLC	Biopsy	pT2a	N0	M0
12	m	79	Inflammatory myofibroblastic tumor	Surgery			
13	m	69	SCLC	Biopsy	cT4	cN3	cM1c
14	f	71	NSCLC	Surgery	pT2a	pN0	cM0
15	f	73	NSCLC	Surgery	pT1b	pN0	cM0
16	m	77	Malign	Interdisciplinary Tumor board	cT1b	N0	M0
17	f	76	NET	Surgery	pT2a	pN0	pM0
18	f	41	Benign	Interdisciplinary Tumor board			
20	m	69	NSCLC	Surgery	pT1b	pN0	pM0
21	f	57	NSCLC	Surgery	pT1c	pN0	pM0
22	f	56	Hamartoma	Follow-up			
23	f	56	Sarcoidosis	Biopsy			
24	f	73	Regredient Lesion	Interdisciplinary Tumor board			
25	f	57	NSCLC	Biopsy	cT3c	cN1	pM1a
26	m	52	Inflammation	Follow-up			
28	m	61	Primary Lung Tumor	Interdisciplinary Tumor board	cT1b	cN0	cM0
29	m	66	Primary Lung Tumor	Interdisciplinary Tumor board	cT4	cN2	cM1b
30	f	69	NSCLC	Biopsy	cT4	cN0	cM0
31	m	59	NSCLC	Surgery	pT2b	pN0	cM0
32	f	54	NSCLC	Surgery	pT2a pT1a	pN1 pN0	pMx pMx
33	m	73	NSCLC	Biopsy	T2b	Nx	M1
35	m	54	Inflammation	Biopsy			
36	m	65	NSCLC	Biopsy	cT2a	cN2	cM0

CLL = Chronic Lymphatic Leukemia; NET = Neuroendocrine Tumor; NSCLC = Non-Small Cell Lung Cancer; SCLC = Small Cell Lung Cancer.

**Table 2:** Measurements of lung lesions, lymph nodes, and metastases depending on their classification as benign, malignant, or inflammatory.

	Total	Malign	Benign	Inflammation
**Lung lesions**	n = 32	n = 21	n = 6	n = 5
Volume (mL)	33.41 ± 58.63	48.34 ± 67.09	1.85 ± 1.71 *	8.63 ± 5.80 *
Density (HU)	19.55 ± 28.93	20.78 ± 30.18	3.72 ± 23.26 *	33.40 ± 25.16
SUV_mean_	6.45 ± 5.56	8.40 ± 5.89	2.05 ± 1.33 *	3.50 ± 1.89
Patlak Ki_mean_ (mL/(min × 100 mL))	1.93 ± 2.1	2.67 ± 2.26	0.30 ± 0.17 *	0.78 ± 0.56
MR-FDG_mean_ (µmol/(min × 100 mL))	10.82 ± 12.62	15.01 ± 13.68	1.56 ± 0.80 *	3.88 ± 3.38
DV-FDG_mean_ (%)	110.35 ± 99.56	114.25 ± 106.95	31.13 ± 9.87 *	63.03 ± 26.89
**Lymph nodes**	n = 65	n = 6	n = 47	n = 12
Short-axis (mm)	9.38 ± 5.75	17.73 ± 8.22	7.61 ± 2.73 *	6.57 ± 0.54 *
Long-axis (mm)	15.95 ± 7.97	26.52 ± 10.35	12.32 ± 4.27 *	12.45 ± 3.93 *
Volume (mL)	2.05 ± 6.40	8.17 ± 13.63	0.65 ± 7.22	0.77 ± 0.66
SUV_mean_	3.43 ± 4.60	11.09 ± 6.54	1.67 ± 0.68 *	1.86 ± 0.38 *
Patlak Ki_mean_ (mL/(min × 100 mL)	0.70 ± 1.14	2.47 ± 1.80	0.28 ± 0.16 *	0.40 ± 0.69 *
MR-FDG_mean_ (µmol/(min × 100 mL))	3.85 ± 6.58	14.31 ± 10.13	1.50 ± 0.83 *	1.32 ± 0.30 *
DV-FDG_mean_ (%)	57.02 ± 36.00	112.69 ± 44.81	43.13 ± 17.24 *	54.50 ± 12.35 *
**Metastases**		n = 7		
SUV_mean_		6.94 ± 4.00		
Patlak Ki_mean_ (mL/(min × 100 mL))		1.47 ± 1.03		
MR-FDG_mean_ (µmol/(min × 100 mL))		8.37 ± 5.82		
DV-FDG_mean_ (%)		69.45 ± 49.63		

* The asterisk and bold font reflects the significant result (*p* < 0.01) of Tukey’s honestly significant difference procedure for multiple comparison correction when separately comparing benign and inflammation to malign findings. One-way ANOVA was significant for main group effects in all evaluations (*p* < 0.05). HU: Hounsfield Units.

**Table 3:** AUC values of pulmonary lesions (n = 32, prevalence: 52.4%).

	AUC	Std. Error	95% CI	*p*-Value	Cut-off Value	Sens.	Spez.
**PET: SUV_mean_**	**0.827**	**0.073**	**0.684–0.970**	**0.003**	**3.08**	**81.0%**	**72.7%**
**PET: MR-FDG_mean_**	**0.887**	**0.057**	**0.775–1.000**	**<0.001**	**61.7** (µmol/(min × 100 mL))	**81.0%**	**81.8%**
**PET: Patlak Ki-FDG_mean_**	**0.861**	**0.065**	**0.735–0.988**	**0.001**	**0.68** (mL/(min × 100 mL))	**81.0%**	**81.8%**
**PET: DV-FDG_mean_**	**0.818**	**0.075**	**0.671–0.965**	**0.004**	**54.3%**	**76.2%**	**81.8%**
**Ratio: SUV_mean_ lesion/SUV_mean_ blood pool**	**0.835**	**0.070**	**0.698–0.973**	**0.002**	**1.86**	**71.4%**	**72.7%**
**Ratio: SUV_mean_ lesion/SUV_mean_ liver tissue**	**0.838**	**0.071**	**0.699–0.977**	**0.002**	**1.38**	**71.4%**	**72.7%**
**CT: Lesion volume**	**0.797**	**0.078**	**0.643–0.950**	**0.007**	**5.6 mL**	**71.4%**	**72.7%**
**CT: Lesion density**	0.550	0.109	0.335–0.764	0.648	17.0 HU	61.9%	63.6%
**CT: Lesion SD density**	0.677	0.103	0.475–0.880	0.104	15.1 HU	76.2%	63.2%

Significant results are highlighted in bold.

**Table 4:** AUC values of mediastinal lymph nodes (n = 65, prevalence: 18.5%).

	AUC	Std. Error	95% CI	*p*-Value	Cut-off Value	Sens.	Spez.
**PET: SUV_mean_**	**0.993**	**0.007**	**0.979–1.000**	**<0.001**	**2.61**	**100%**	**94.3%**
**PET: MR-FDG_mean_**	**0.987**	**0.011**	**0.966–1.000**	**<0.001**	**2.58** (µmol/(min × 100 mL))	**91.7%**	**90.6%**
**PET: Patlak Ki-FDG_mean_**	**0.958**	**0.034**	**0.891–1.000**	**<0.001**	**0.49** (mL/(min × 100 mL))	**83.3%**	**92.5%**
**PET: DV-FDG_mean_**	**0.948**	**0.028**	**0.893–1.000**	**<0.001**	**60.5 %**	**83.3%**	**81.1%**
**CT: short axis**	**0.969**	**0.020**	**0.929–1.000**	**<0.001**	**10.5 mm**	**91.7%**	**84.9%**
**CT: long axis**	**0.947**	**0.028**	**0.893–1.000**	**<0.001**	**16.1 mm**	**83.3%**	**84.9%**

Significant results are highlighted in bold.

## Data Availability

The data presented in this study are available on request from the corresponding author. The data are not publicly available due to data protection regulations.

## Supplementary Materials

The following supporting information can be downloaded at: https://www.mdpi.com/article/10.3390/jcm12123942/s1. Table S1: Extended AUC values of pulmonary lesions (n = 32, prevalence: 52.4%). Table S2: Extended AUC values of thoracic lymph nodes (n = 65, prevalence: 18.5%).

## References

[1] Barta J.A., Powell C.A., Wisnivesky J.P. (2019). Global Epidemiology of Lung Cancer. Ann. Glob. Health.

[2] Wankhede D. (2021). Evaluation of Eighth AJCC TNM Sage for Lung Cancer NSCLC: A Meta-analysis. Ann. Surg. Oncol..

[3] Postmus P.E., Kerr K.M., Oudkerk M., Senan S., Waller D.A., Vansteenkiste J., Escriu C., Peters S. (2017). Early and locally advanced non-small-cell lung cancer (NSCLC): ESMO Clinical Practice Guidelines for diagnosis, treatment and follow-up. Ann. Oncol..

[4] Kalemkerian G.P., Loo B.W., Akerley W., Attia A., Bassetti M., Boumber Y., Decker R., Dobelbower C., Dowlati A., Grecula J.C. (2018). NCCN Guidelines Insights: Small Cell Lung Cancer, Version 2.2018. J. Natl. Compr. Cancer Netw..

[5] Coello C., Fisk M., Mohan D., Wilson F.J., Brown A.P., Polkey M.I., Wilkinson I., Tal-Singer R., Murphy P.S., Cheriyan J. (2017). Quantitative analysis of dynamic (18)F-FDG PET/CT for measurement of lung inflammation. EJNMMI Res..

[6] Yang M., Lin Z., Xu Z., Li D., Lv W., Yang S., Liu Y., Cao Y., Cao Q., Jin H. (2020). Influx rate constant of (18)F-FDG increases in metastatic lymph nodes of non-small cell lung cancer patients. Eur. J. Nucl. Med. Mol. Imaging.

[7] Shen G., Hu S., Deng H., Jia Z. (2015). Diagnostic value of dual time-point 18 F-FDG PET/CT versus single time-point imaging for detection of mediastinal nodal metastasis in non-small cell lung cancer patients: A meta-analysis. Acta Radiol..

[8] Nogami Y., Banno K., Irie H., Iida M., Masugi Y., Murakami K., Aoki D. (2015). Efficacy of 18-FDG PET-CT dual-phase scanning for detection of lymph node metastasis in gynecological cancer. Anticancer Res..

[9] Shinya T., Rai K., Okumura Y., Fujiwara K., Matsuo K., Yonei T., Sato T., Watanabe K., Kawai H., Sato S. (2009). Dual-Time-Point F-18 FDG PET/CT for Evaluation of Intrathoracic Lymph Nodes in Patients With Non-Small Cell Lung Cancer. Clin. Nucl. Med..

[10] Karakatsanis N.A., Lodge M.A., Tahari A.K., Zhou Y., Wahl R.L., Rahmim A. (2013). Dynamic whole-body PET parametric imaging: I. Concept, acquisition protocol optimization and clinical application. Phys. Med. Biol..

[11] Karakatsanis N.A., Lodge M.A., Zhou Y., Wahl R.L., Rahmim A. (2013). Dynamic whole-body PET parametric imaging: II. Task-oriented statistical estimation. Phys. Med. Biol..

[12] Rahmim A., Lodge M.A., Karakatsanis N.A., Panin V.Y., Zhou Y., McMillan A., Cho S., Zaidi H., Casey M.E., Wahl R.L. (2019). Dynamic whole-body PET imaging: Principles, potentials and applications. Eur. J. Nucl. Med. Mol. Imaging.

[13] Dias A.H., Pedersen M.F., Danielsen H., Munk O.L., Gormsen L.C. (2021). Clinical feasibility and impact of fully automated multiparametric PET imaging using direct Patlak reconstruction: Evaluation of 103 dynamic whole-body (18)F-FDG PET/CT scans. Eur. J. Nucl. Med. Mol. Imaging.

[14] Patlak C.S., Blasberg R.G. (1985). Graphical evaluation of blood-to-brain transfer constants from multiple-time uptake data. Generalizations. J. Cereb. Blood Flow Metab..

[15] Patlak C.S., Blasberg R.G., Fenstermacher J.D. (1983). Graphical evaluation of blood-to-brain transfer constants from multiple-time uptake data. J. Cereb. Blood Flow Metab..

[16] Smith A.M., Spottiswoode B.S., Vijay, Hu J., von Gall C. (2018). FlowMotion Multiparametric PET Suite—The Patlak Model.

[17] Ye Q., Wu J., Lu Y., Naganawa M., Gallezot J.D., Ma T., Liu Y., Tanoue L., Detterbeck F., Blasberg J. (2018). Improved discrimination between benign and malignant LDCT screening-detected lung nodules with dynamic over static (18)F-FDG PET as a function of injected dose. Phys. Med. Biol..

[18] Dias A.H., Hansen A.K., Munk O.L., Gormsen L.C. (2022). Normal values for (18)F-FDG uptake in organs and tissues measured by dynamic whole body multiparametric FDG PET in 126 patients. EJNMMI Res..

[19] Laffon E., Marthan R. (2021). Is Patlak y-intercept a relevant metrics?. Eur. J. Nucl. Med. Mol. Imaging.

