# Supplementary material for: Diagnostic Performance of Dynamic Whole-Body Patlak [18F]FDG-PET/CT in Patients with Indeterminate Lung Lesions and Lymph Nodes
Source: J Clin Med. 2023 Jun 9;12(12):3942. doi: 10.3390/jcm12123942 (PMC10299392; doi:10.3390/jcm12123942)
Supplement: Supplementary file 1 [file jcm-12-03942-s001.zip › jcm-2358658-supplementary.pdf]

Supplementary Table S1. Extended AUC values of pulmonary lesions (n=32, prevalence: 52.4%).

	AUC	Std.Error	95% CI	p-value
**SUVmean**	0.827	0.073	0.684 - 0.970	0.003
**SUVmax**	0.823	0.075	0.676 – 0.969	0.003
**SUVpeak**	0.861	0.066	0.731 – 0.992	0.001
**MR-FDGmean**	0.887	0.057	0.775-1.000	<0.001
**MR-FDGmax**	0.887	0.058	0.774 – 1.000	<0.001
**MR-FDGpeak**	0.896	0.055	0.789 – 1.000	<0.001
**Patlak Ki-FDGmean**	0.861	0.065	0.735-0.988	0.001
**Patlak Ki-FDGmax**	0.855	0.066	0.726 – 0.984	0.001
**Patlak Ki-FDGpeak**	0.872	0.063	0.750 – 0.995	0.001
**DV-FDGmean**	0.818	0.075	0.671-0.965	0.004
**DV-FDGmax**	0.816	0.075	0.669 – 0.963	0.004
**DV-FDGpeak**	0.831	0.073	0.689 – 0.974	0.002

Supplementary Table S2. Extended AUC values of thoracic lymph nodes (n=65, prevalence: 18.5%).

	AUC	Std.Error	95% CI	p-value
**SUVmean**	0.993	0.007	0.979 – 1.000	< 0.001
**SUVmax**	0.995	0.006	0.984 – 1.000	< 0.001
**SUVpeak**	0.995	0.006	0.984 – 1.000	< 0.001
**MR-FDGmean**	0.987	0.011	0.966 – 1.000	< 0.001
**MR-FDGmax**	0.994	0.007	0.980 - 1.000	< 0.001
**MR-FDGpeak**	0.991	0.009	0.974 – 1.000	< 0.001
**Patlak Ki-FDGmean**	0.958	0.034	0.891 – 1.000	< 0.001
**Patlak Ki-FDGmax**	0.984	0.012	0.961 – 1.000	< 0.001
**Patlak Ki-FDGpeak**	0.977	0.016	0.945 – 1.000	< 0.001
**DV-FDGmean**	0.948	0.028	0.893 – 1.000	< 0.001
**DV-FDGmax**	0.975	0.016	0.943 – 1.000	< 0.001
**DV-FDGpeak**	0.972	0.018	0.938 – 1.000	< 0.001
